# Two-Dimensional Analysis of Horizontal and Vertical Pursuit in Infantile Nystagmus Reveals Quantitative Deficits in Accuracy and Precision

**DOI:** 10.1167/iovs.61.6.15

**Published:** 2020-06-11

**Authors:** Lee Mcilreavy, Tom C. A. Freeman, Jonathan T. Erichsen

**Affiliations:** 1 School of Optometry and Vision Sciences, Cardiff University, Cardiff, United Kingdom; 2 School of Psychology, Cardiff University, Cardiff, United Kingdom

**Keywords:** smooth pursuit, eye velocity, gaze holding, pursuit gain, probability density function

## Abstract

**Purpose:**

Infantile nystagmus (IN) presents with continuous, predominantly horizontal eye oscillations. It remains controversial whether those with IN have normal horizontal pursuit, while vertical pursuit has rarely been studied. We examined whether there are pursuit deficits associated with IN by investigating the effect of target direction, velocity, and amplitude.

**Methods:**

Twelve adults with idiopathic IN performed a pursuit task, a 0.4° dot moved either horizontally or vertically at 8 or 16°/s, through amplitudes of 8°, 16°, or 32°. Accuracy and precision errors were computed as bivariate probability density functions of target-relative eye velocities.

**Results:**

Eye velocity was less precise along the horizontal axis during both horizontal and vertical pursuit, reflecting the primary axis of the eye oscillation. Mean accuracy error along the target trajectory during vertical pursuit was just as impaired as during horizontal pursuit. There was a greater error in accuracy along the target trajectory for 16°/s targets than 8°/s. Finally, targets that oscillated at 2.0 Hz had a greater error in accuracy along the target trajectory than frequencies of 1.0 Hz or 0.5 Hz. When studied using the same experimental protocol, pursuit performance for typical observers was always better.

**Conclusions:**

These findings strongly support our hypothesis of severe deficits in pursuit accuracy in observers with IN for horizontally and vertically moving targets, as well as for targets that move at higher speeds or oscillate more quickly. Overall, IN pursuit impairment appears to have previously been underestimated, highlighting a need for further quantitative studies of dynamic visual function in those with IN.

Infantile nystagmus presents with continuous eye oscillations that are predominantly horizontal.^1^ The condition manifests in the first few months following birth and is lifelong with no known cure. The condition is relatively common with a prevalence of 14 per 10,000 of the population^2^ and significantly impacts on the quality of life of those affected.^3^^,^^4^ Indeed, nystagmus and other irregular eye movements represent the second most common cause (11%) for children to be registered as sight impaired in England and Wales.^5^ Infantile nystagmus is associated with many conditions (e.g., albinism),^6^ but the etiology of the eye oscillation is still unknown. Pursuit involves many brain centers, from brainstem to cortex.^7^ One current model of the condition argues that the oscillation is generated by an antagonism between the accessory optic system and an intact, yet normal, cortical pursuit system,^8^ although this model has not yet been tested.

Pursuit eye movements are considered gaze shifting,^9^ enabling small moving objects to be continuously imaged on, or in close proximity to, the fovea for detailed inspection.^10^^,^^11^ Despite their reduced visual acuity and ongoing eye oscillation, one major theoretical issue that has dominated the field of infantile nystagmus research is whether those with the condition can follow moving targets with an appropriate eye velocity.

Early studies of optokinetic nystagmus (OKN) and pursuit in those with infantile nystagmus calculated the gain of the eye movements (i.e., the ratio of eye velocity to target velocity) using the entire distribution of slow-phase eye velocities and reported lower gains for both types of eye movement when compared to typical observers.^12^^–^^14^ Subsequent studies differed in how gain was calculated,^15^^,^^16^ with these authors arguing that pursuit eye velocity was superimposed on the slow-phase velocity. They reasoned that pursuit gain should only be estimated during foveation periods, when the overall eye velocity would have a relatively small nystagmus component. In such analyses, the authors linearly interpolated between successive foveation periods on a position-time graph and the slope of the fitted line regarded as the pursuit eye velocity. Using this approach, these studies have indicated that those with pursuit have normal gains, a finding that has been used as a basis for recent models of infantile nystagmus.^8^ However, it is important to realize that the calculated accuracy depends on the proportion of slow-phase velocity that is analyzed. For instance, if the proportion of eye movements used corresponded only to those periods when the eyes were fixating the target, the gain would inevitably be 1.

Although many researchers have examined horizontal pursuit in those with infantile nystagmus, relatively little is known about vertical pursuit in those with this condition. Qualitative descriptions suggest those with infantile nystagmus have “normal” vertical pursuit performance.^17^ Indeed, these descriptions would seem to be supported by studies showing vertical OKN gains within the normal range.^12^ However, the results of vertical OKN studies cannot necessarily be extrapolated to vertical pursuit eye movements owing to differences in the stimuli used, especially their size, as well as the underlying mechanisms involved. Although the infantile nystagmus oscillation produces a predominantly horizontal eye movement, a large, horizontally oriented luminance grating ensures that the OKN stimulus remains imaged on the fovea. However, the same horizontal eye movement would cause a small vertically moving pursuit stimulus to be imaged extrafoveally most of the time.

To date, in almost all studies, pursuit success has exclusively been confined to the analysis of the eye movement component parallel to the target and has emphasized accuracy (i.e., average eye velocity) rather than precision (i.e., the variability of eye velocities).^18^ The implicit assumption is that eye velocities perpendicular to the target motion are accurate and precise. Yet, studies of infantile nystagmus have shown significant eye movement components orthogonal to the pursuit target.^1^ Limiting the analysis to a one-dimensional analysis may therefore underplay the complexities of pursuit in infantile nystagmus.

Previous work has suggested that those with infantile nystagmus can follow rapidly oscillating horizontal pursuit targets with greater success than typical individuals.^19^ However, that study used a qualitative one-dimensional analysis of a single participant with pendular nystagmus and so is open to question. Therefore, the main motivation of the current study is to better understand the two-dimensional nature of pursuit in infantile nystagmus. We used a novel two-dimensional analysis based on the velocity error between the eye and target^20^ to investigate the influence of target direction and velocity on pursuit performance, as well as on oscillation frequency and amplitude.

## Materials and Methods

### Participants

This investigation was carried out in accordance with the Declaration of Helsinki. Informed consent was obtained from all observers after they received an explanation of the nature and possible consequences of the study. Ethical approval was granted by the School Research Ethics Committee at the School of Optometry and Vision Sciences, Cardiff University. Twelve adult observers with idiopathic infantile nystagmus volunteered for this study (mean 47.5 ± 13.9 years; range 18–69 years; seven male and five female). After a detailed family history, all observers had their diagnosis confirmed using high-speed eye movement recording, slit-lamp examination, ophthalmoscopy, and optical coherence tomography (OCT).

### Materials

All experiments took place within a dark room. Monocular eye position was recorded using a tower-mounted EyeLink 1000 (SR-Research, Ottawa, Ontario, Canada), with the head supported by a chin and forehead rest. Observers wore their habitual refractive correction, if any, and the eye with better acuity was used for recording, with the contralateral eye occluded by an eye patch. In the case of equal acuities, the right eye was used by default. Stimuli were rear projected using a CRT projector (Multiscan VPH 1272QM; Sony, Tokyo, Japan) onto a large screen (200 × 155 cm) that was positioned 140 cm from the observer. All stimuli were generated using the OpenGL graphics library driven by the Delphi programming environment (version 7; Borland Software Company, Cupertino, CA, USA) and displayed using a GeForce 7300 LE graphics card (NVIDIA, Santa Clara, CA, USA) at a frame rate of 72 Hz and a resolution of 1024 by 768 pixels.

### Stimuli

The pursuit target was a 0.4° dot moving at either 8°/s or 16°/s and with a peak-to-peak amplitude of 8°, 16°, or 32°. Pursuit was along two planes, either horizontal (left-right pursuit) or vertical (up-down pursuit).

### Procedure

All observers were instructed to follow the target to the best of their ability. Each trial was initiated by a single button press on a wireless keyboard, which caused the target to initially step (in the direction opposite to the subsequent pursuit) by half the pursuit amplitude and then remained stationary for 2 seconds before continuously moving with a triangular waveform. Trials were arranged into four blocks, with two blocks for each pursuit direction. Thus, each block contained six trials (2 velocities × 3 amplitudes). The order of the blocks and the respective trials was randomized for each observer. Eye movements were calibrated prior to each block. Calibration targets were stationary dot targets (0.4°) equally spaced (16°) and arranged in a 3 × 3 grid. Observers sequentially viewed each calibration target for 10 seconds, and only the slowest 10% of each nystagmus slow phase was used for calibration.^21^

### Eye Movement Analysis

Eye movement data were analyzed offline using scripts written in MATLAB (version 2016b; MathWorks, Natick, MA, USA). The initial 2 seconds of data, when the target was stationary, were discarded. Eye position data from the central 70% of each sweep of the target were used in the subsequent analysis to avoid pursuit initiation artifacts and anticipatory slowing of eye movements associated with the reversal of target direction. Eye movement data for repeated trials were concatenated before they were parsed into their respective pursuit directions (right, left, up, down).

Eye position data were filtered using a fourth-order Butterworth filter with a 60-Hz cutoff, prior to temporal differentiation to obtain velocity, acceleration, and jerk. Artifacts, representing blinks and sporadic dropped data, were identified as those regions where jerk exceeded an arbitrary threshold of 3 × 10^6^ deg/s^3^. Saccades and fast phases were identified in the remaining data and removed using a velocity criterion (mean eye velocity plus a multiple of the standard deviation) that was unique to each observer and adjusted by the experimenter. The thresholds for artifact and saccade detection were based on multiple observations and adjusted by the experimenter (author L.M.) to ensure accurate identification. We did not undertake an analysis of the fast phases of infantile nystagmus since their role during pursuit has already been shown to be compensatory, redirecting gaze back on to the target.^22^ Further, the trajectory corresponding to the end position of the fast phases follows the trajectory of the target.^22^

The method of analyzing the remaining velocity samples (i.e., slow phases) is reported in detail elsewhere.^20^ Briefly, two-dimensional velocity error distributions were derived by expressing slow-phase velocities relative to the pursuit target. Bivariate probability density functions were then computed on the velocity error distributions using an open source script.^23^

The isocontour that encompassed 68% of the highest probability density velocity error data was selected for further analysis. We quantified the accuracy error of eye velocity using the x and y coordinates of the isocontour centroid. Since the origin of the velocity error distribution represents the target velocity, larger coordinate values indicate larger errors in eye velocity accuracy. Precision error was quantified using the area of the isocontour, from which the major and minor axes of the equivalent ellipse were calculated. In the case of precision, larger values for major and minor axes indicate larger error in eye velocity precision. The angle between the horizontal axis and the major axis of the velocity isocontour was analyzed to determine the major axis of the velocity error during pursuit.

### Statistical Analysis

Angular data (i.e., orientations of the isocontour major axes) were analyzed with circular statistics using an open source toolbox for MATLAB.^24^ A Kuiper's *V* test was used to statistically determine whether angular data were distributed uniformly around a circle (null hypothesis) or had an assumed mean direction (alternative hypothesis). To determine the effect of target direction, velocity, and amplitude on accuracy and precision, a three-factor repeated-measures ANOVA was performed. Mauchly's test for sphericity was performed with each ANOVA, and when the assumption of sphericity had been violated, the degrees of freedom were adjusted using a Greenhouse-Geisser correction. An alpha level of 0.05 was used to determine statistical significance, and a Bonferroni correction for multiple comparisons was applied to all post hoc tests. All repeated-measure ANOVAs were carried out using JASP software (version 0.9.2; JASP Team, Amsterdam, The Netherlands). All error bars represent the 95% confidence intervals, calculated using the Cousineau-Morey method.^25^^,^^26^

## Results

**Figure 1.**
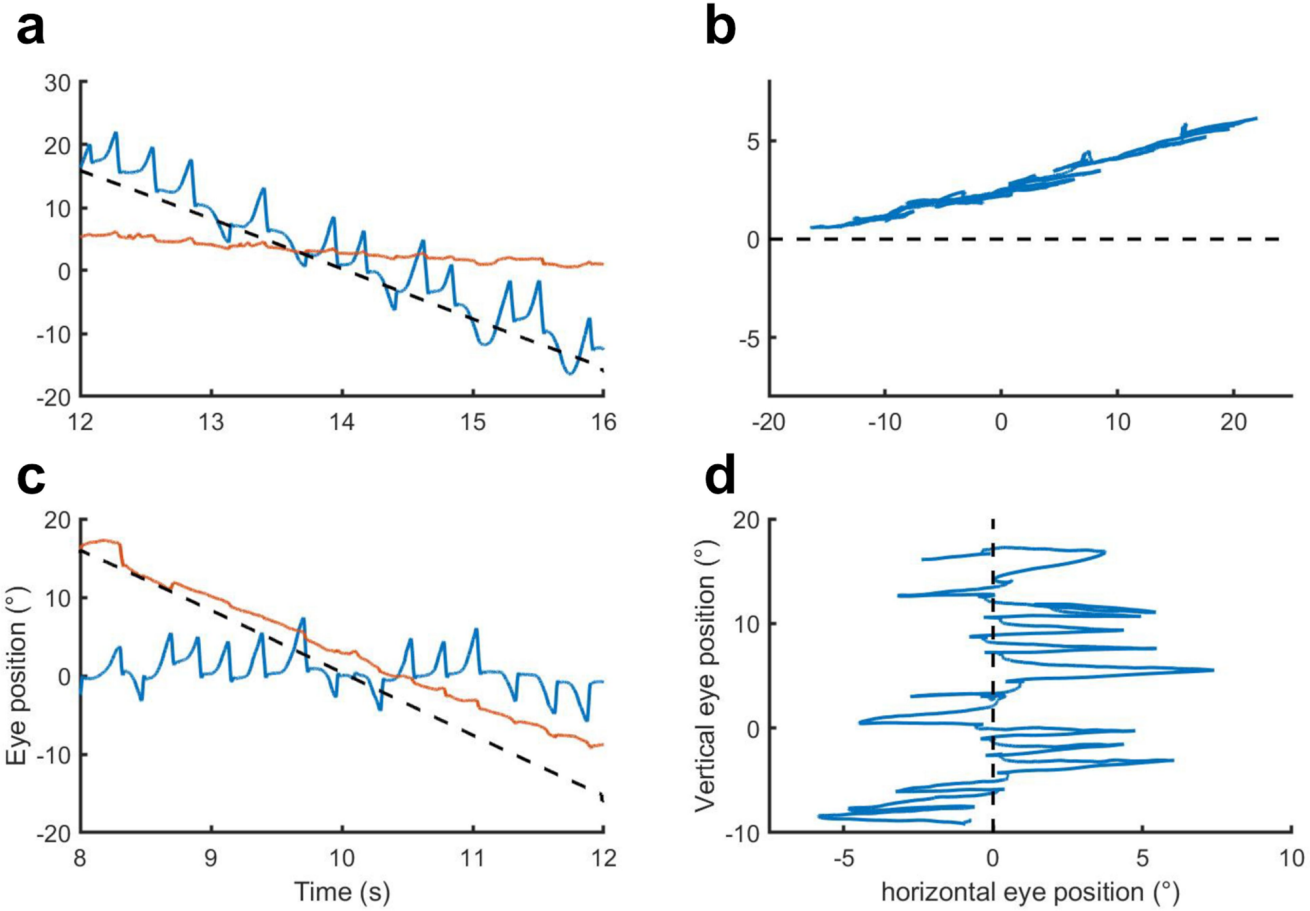
Representative sample of two-dimensional eye position during pursuit (data from observer 03). (**a**) A position-time plot of horizontal (*blue trace*) and vertical (*orange trace*) eye position during horizontal pursuit. The target trajectory (*black dashed line*) was leftward with an amplitude of 36° at 8°/s. This plot shows the observer attempting to follow the target's horizontal trajectory despite the ongoing horizontal eye oscillation. In each cycle, the slow phases move gaze away from the moving target until a fast phase redirects eye position back onto the target. The corresponding two-dimensional plot of eye movements for these data is shown (**b**) and demonstrates the primary axis of the oscillation is collinear with the target trajectory. (**c**) Similar to (**a**), this is a position-time plot of horizontal and vertical eye position during vertical pursuit. The pursuit trajectory was downward with an amplitude of 36° at 8°/s. This plot shows the observer attempting to follow the targets’ vertical trajectory despite an ongoing horizontal eye oscillation. The corresponding two-dimensional plot of the eye movements for these data is shown (**d**). Note that unlike (**b**), the primary axis of the eye oscillation is perpendicular to the target trajectory.

**Figure 2.**
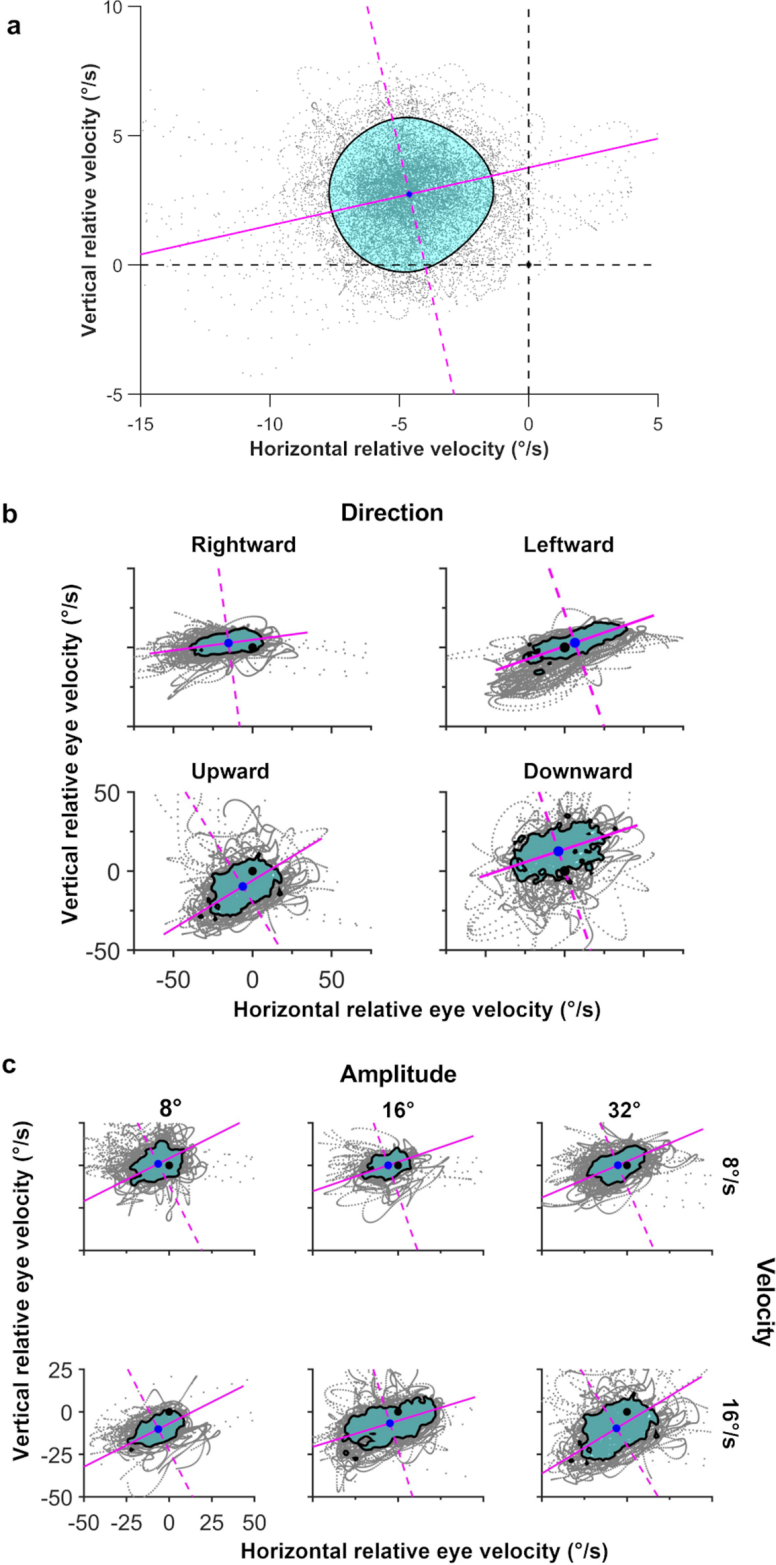
(**a**) Schematic summary of the metrics used to quantify pursuit performance in those with infantile nystagmus. Eye velocity samples (*gray* *data*) were plotted relative to the target velocity. Thus, the origin of the velocity error distribution is the target velocity. A bivariate probability density function was computed on the eye velocity samples, and the contour that encompassed the highest 68% of probability densities was selected for further analysis (*black contour*). The magnitude of the coordinates of the centroid of this contour (*blue dot*) from the origin (i.e., the target velocity) is a measure of the accuracy of eye velocity. Larger coordinate values represent lower accuracy. The area within the 68% isocontour represents the overall precision of the eye velocity during pursuit. In this study, we expressed the precision along the major (*solid magenta line*) and minor axes (*dashed magenta line*) using an equivalent ellipse with the same area as the isocontour. Finally, we measured the orientation of the major axis, relative to the horizontal axis using the four-quadrant inverse tangent. Note that these orientation data are axial, such that a vertical line lies along the 90° to 270° orientation, whereas a horizontal line lies along the 0° to 180° orientation. (**b**) Representative sample of results for the main effect of target direction (16°/s across 32°). (**c**) Representative sample of results for the main effect of velocity and amplitude (vertical pursuit). All data in (**a**), (**b**), and (**c**) were taken from observer 06. The format of each isocontour figure is the same as (**a**). Although there was variability between individual observers with infantile nystagmus, the results for the infantile nystagmus cohort indicate that the major axis (*solid magenta line*) was, on average, oriented horizontally, for both horizontal and vertical pursuit. However, the mean orientation was more variable during vertical pursuit. Vertical pursuit was just as impaired (i.e., low accuracy and low precision) as horizontal pursuit along the target trajectory. The higher target velocity resulted in less accurate and less precise pursuit along the target trajectory. Finally, a smaller target amplitude resulted in less accurate pursuit along the target trajectory.

Representative samples of two-dimensional eye position data from an observer with infantile nystagmus during a horizontal and vertical pursuit trial are shown in Figure 1, while representative velocity isocontours for each of the target manipulations (direction, velocity, and amplitude) are shown in Figure 2. In each plot of Figure 2, eye velocity samples (gray) are plotted relative to the target. Superimposed on these eye velocity data is the eye velocity isocontour (cyan area) that corresponds to the highest 68% probability density. The solid and dashed magenta lines are always orthogonal to one another and indicate the major and minor axes of the velocity isocontour. Thus, the major axis indicates the orientation of the greatest spread of eye velocities (greater error in eye velocity precision), whereas the minor axis indicates the orientation of least spread (lower error in eye velocity precision). Both the major and minor axes intersect at the center of mass of the velocity isocontour (blue dot). The greater the distance of the isocontour centroid from the origin (black dot) of the graph, the greater the error in eye velocity accuracy.

### Major Axis Orientation

**Figure 3.**
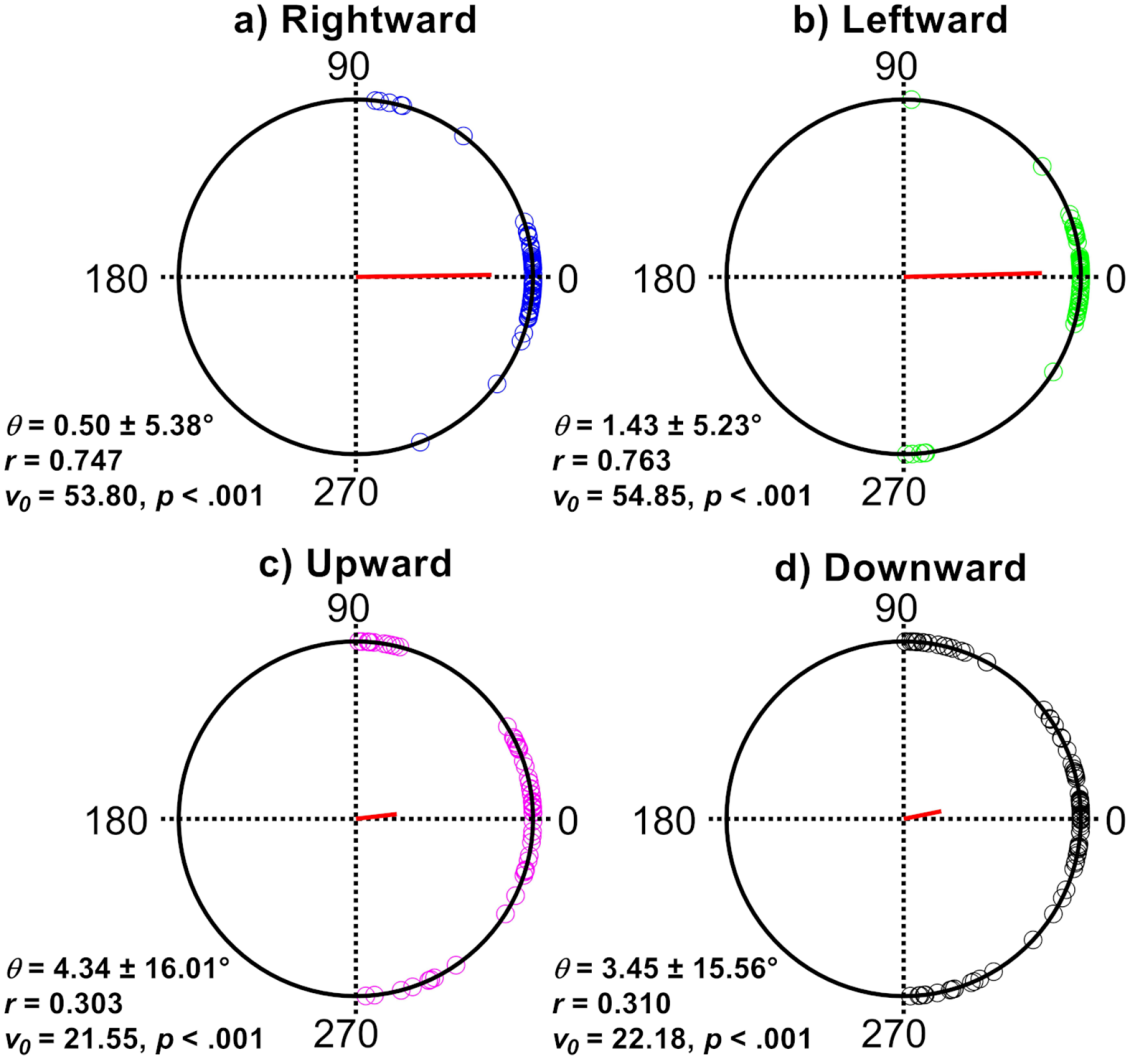
Polar distributions of the major axes of the velocity isocontours. Each unfilled circle represents the result of a single pursuit trial, with each direction having 72 individual data points (2 velocities × 3 amplitudes × 12 observers). The resultant vector (indicated by the *red line*) of each distribution has both orientation (*Ɵ*) and magnitude (*r*). The orientation of the resultant vector is the mean angle for the distribution, whereas its magnitude (length) indicates how concentrated the distribution is. The closer the resultant vector approaches the unit circle (a value of 1), the more concentrated are the angular data. Kuiper's *V* test result (v_α_) indicates whether the distribution had a significant mean direction (α). In all cases, the major axis was aligned horizontally. The magnitude of the resultant vector was larger for horizontal pursuit directions, indicating less circular spread of data. Indeed, the 95% confidence intervals for vertical pursuit were almost an order of magnitude tighter than for horizontal pursuit.

We first analyzed the orientations of the isocontour major axes as a function of target direction. Since the predominant axis of the nystagmus oscillation is horizontal, we predicted that, whatever the direction of the pursuit target, the error in eye velocity precision would be greatest along a horizontal orientation. If our prediction was correct, then the major axes of the velocity isocontours should align approximately horizontally. Figure 3 shows polar distributions of the major axis orientation for each pursuit direction.

Each plot contains 72 data points (1 direction × 2 velocities × 3 amplitudes × 12 observers). The red line extending from the center of each plot is the resultant vector, which has both orientation (*Ɵ*) and length (*r*). The orientation indicates the mean orientation of the underlying data, while the length indicates how concentrated the data are at an orientation. The closer the resultant vector is to the unit circle (i.e., as *r* approaches 1), the more concentrated the data.

For horizontal pursuit directions, the mean resultant vector has an orientation of less than 1.5°, suggesting that the underlying data are orientated horizontally. In both rightward and leftward pursuit data, the length of the resultant vector exceeds 0.74, suggesting that the underlying data are concentrated at this orientation. Using the Kuiper’s *V* test, we tested whether these horizontal pursuit data have no mean orientation (*H_0_*) or an a priori mean orientation (*H_1_*) corresponding to 0° (i.e., horizontal). The results indicated that both had an orientation not significantly different from 0° (rightward pursuit, *P* < 0.001; leftward pursuit, *P* < 0.001).

For vertical pursuit, we obtained similar results. The mean resultant vector had an orientation of <4.5°, but the length of the vector was <0.31. This indicated that, while on average the data were aligned horizontally, there was considerable spread. Once again, we tested whether the data had an a priori mean orientation of 0°, with results showing both directions were not significantly different from 0° (upward pursuit, *P* < 0.001; downward pursuit, *P* < 0.001).

The results for the orientation of the major axis confirmed our first prediction that eye velocity is, on average, more variable along the axis of the nystagmus oscillation (i.e., horizontally), regardless of whether the target moved up, down, left, or right.

### Effect of Target Direction, Velocity, and Amplitude

The results of the repeated-measures ANOVA for main effects of target direction, velocity, and amplitude on each of the pursuit metrics are presented in Table, along with their interaction effects. It should be noted that, for each main effect for a given independent variable, data have been collapsed across the other independent variables (e.g., main effects for target direction are the result of averaging all trials by direction, across all target velocities and amplitudes).

**Table.** Repeated-Measures ANOVA Results for the Two Dependent Measures, Accuracy and Precision

Characteristic	*df*	*F*	*P*	η^2^
*Accuracy error_A_*				
Direction	1.46, 16.09	2.91	0.096	0.209
Velocity	1.00, 11.00	72.48	<.001***	0.868
Amplitude	2.00, 22.00	9.88	<.001***	0.473
D × V	1.98, 21.76	4.90	0.018*	0.308
D × A	3.44, 37.80	1.30	0.290	0.105
V × A	2.00, 22.00	2.03	0.155	0.156
D × V × A	2.52, 27.73	0.37	0.740	0.033
*Accuracy error_O_*				
Direction	1.70, 18.67	10.08	0.002**	0.478
Velocity	1.00, 11.00	1.96	0.189	0.151
Amplitude	1.24, 13.68	1.76	0.210	0.138
D × V	1.21, 13.31	0.07	0.844	0.006
D × A	1.83, 20.17	0.97	0.390	0.081
V × A	1.14, 12.49	0.10	0.747	0.009
D × V × A	2.14, 23.57	0.89	0.432	0.075
*Precision error_A_*				
Direction	1.34, 14.74	0.77	0.432	0.065
Velocity	1.00, 11.00	9.34	0.011*	0.459
Amplitude	1.29, 14.14	1.67	0.221	0.132
D × V	1.29, 14.21	0.12	0.794	0.011
D × A	1.74, 19.14	1.13	0.336	0.093
V × A	2.00, 22.00	1.06	0.365	0.088
D × V × A	1.85, 20.33	1.89	0.179	0.146
*Precision error_O_*				
Direction	1.29, 14.17	6.56	0.017*	0.373
Velocity	1.00, 11.00	20.35	<.001***	0.649
Amplitude	2.00, 22.00	0.41	0.668	0.036
D × V	1.14, 12.49	5.18	0.038*	0.320
D × A	1.93, 21.27	0.93	0.408	0.078
V × A	2.00, 22.00	3.79	0.039*	0.026
D × V × A	2.52, 27.66	1.95	0.153	0.151

Subscript letters A and O denote measures taken along and orthogonal to the target trajectory, respectively. Significant results are in bold typeface, and the levels of statistical significance are denoted as *P < 0.050, **P < 0.010, or ***P < 0.001.

#### Target Direction

**Figure 4.**
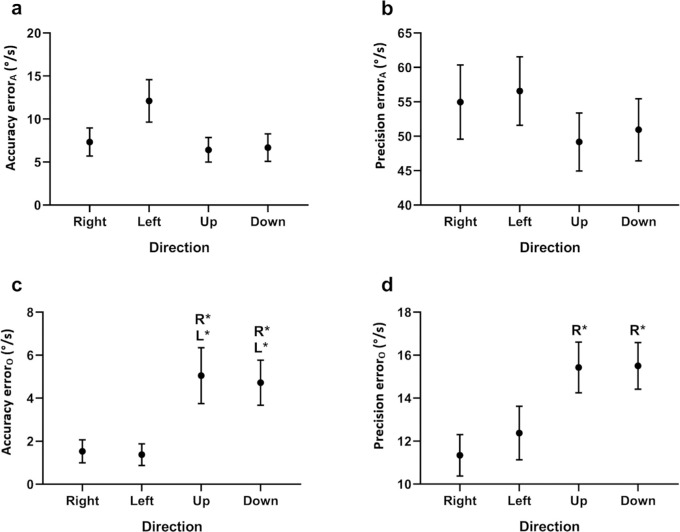
Effect of target direction. Data are the means of the accuracy and precision errors of eye velocity along (**a**, **b**) and orthogonal (**c**, **d**) to the target trajectory. Mean errors for each target direction are the result of averaging across all target velocities and amplitudes. Error bars represent the 95% confidence intervals, calculated using the Cousineau-Morey method.^25^^,^^26^ The significance of post hoc comparisons is displayed above the data. The letters “R” and “L” indicate those results that were significantly different from rightward and leftward target directions, respectively. The levels of statistical significance are denoted as *P < 0.050, **P < 0.010, and ***P < 0.001.

There was no significant main effect of direction on either the accuracy (*P* = 0.096) (Fig. 4a) or precision (*P* = 0.432) (Fig. 4b) of eye velocity along the target trajectory, demonstrating that the eye velocity errors for vertical pursuit are no different from horizontal pursuit. In contrast, there was a significant main effect of direction on both accuracy (*P* = 0.002) (Fig. 4c) and precision (*P* = 0.017) (Fig. 4d) of eye velocity orthogonal to the target trajectory. Post hoc analyses indicated that both horizontal pursuit directions had lower accuracy error than both vertical pursuit directions, but only rightward pursuit had lower precision error than both vertical pursuit directions (see Supplementary Material S1 for statistics).

These results indicate little variation in vertical eye velocity during horizontal pursuit but large variation in horizontal eye velocity during vertical pursuit. This finding is consistent with infantile nystagmus being a predominantly horizontal oscillation with a smaller vertical component^1^ and also with our results for the major axes of the isocontours, which suggested that the horizontal component of eye velocity has a greater precision error, regardless of the target direction.

#### Target Velocity

**Figure 5.**
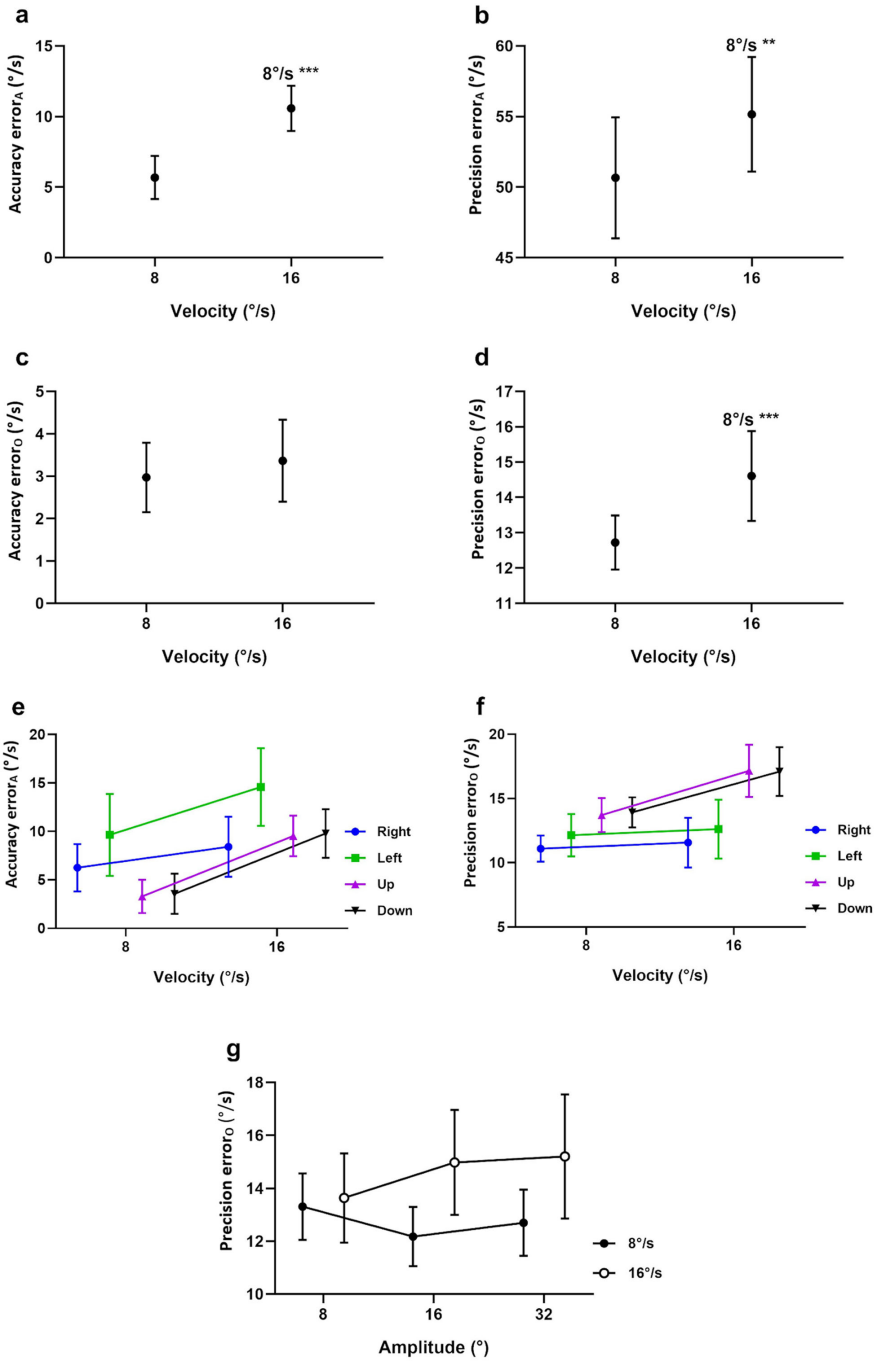
Effect of target velocity (**a–d**). Data are the means of the accuracy and precision errors of eye velocity along (**a**, **b**) and orthogonal (**c**, **d**) to the target trajectory. Mean errors for each target velocity are the result of averaging across all target directions and amplitudes. The significance of post hoc comparisons is displayed above the data. “8°/s” above a result indicates that there was a significance difference from a target velocity of 8°/s. The format and depiction of the levels of statistical significance are the same as Figure 4. Interaction effects for target velocity (**e–f**). Mean accuracy error along the target trajectory as a function of target velocity by direction (**e**). Mean precision error orthogonal to the target trajectory as a function of target velocity by direction (**f**) and target amplitude by velocity (**g**).

There was a significant main effect of target velocity on the accuracy of eye velocity along the target trajectory (*P* < 0.001) (Fig. 5a), with significantly greater error in accuracy at a target velocity of 16°/s than 8°/s (see Supplementary Material S2 for statistics).

This can be explained by the significant interaction between velocity and direction for eye velocity accuracy along the target trajectory (*P* = 0.018) (Fig. 5e). These results suggest that, while the error in accuracy increased for all target directions, there was a greater increase in accuracy error at the higher velocity during vertical (upward, 189% increase; downward, 174% increase) as compared with horizontal pursuit (rightward, 34% increase; leftward, 51% increase).

There was a significant main effect of target velocity on the precision of eye velocity along (*P* = 0.011) (Fig. 5a) and orthogonal (*P* < 0.001) to the target trajectory (Fig. 5d), with significantly higher error in precision in both cases at a target velocity of 16°/s than 8°/s (see Supplementary Material S2 for statistics).

The results for precision orthogonal to the target trajectory can be explained by the significant interaction between velocity and direction (*P* = 0.038) (Fig. 5f), where the error in precision orthogonal to the target trajectory increased considerably at the higher target velocity for vertical pursuit (upward, 25% increase; downward, 23% increase), whereas it remained largely unaffected for horizontal pursuit (rightward, 4% increase; leftward, 4% increase). This result suggests that horizontal eye velocity became more variable during vertical pursuit than vertical eye velocity during horizontal pursuit.

Although there was no significant main effect for amplitude on precision orthogonal to target trajectory (*P* = 0.668), we noted a significant interaction effect for velocity and amplitude (*P* = 0.039), which shows that the difference in precision between the two velocities decreased at the smallest target amplitude (Fig. 5g).

#### Target Amplitude

**Figure 6.**
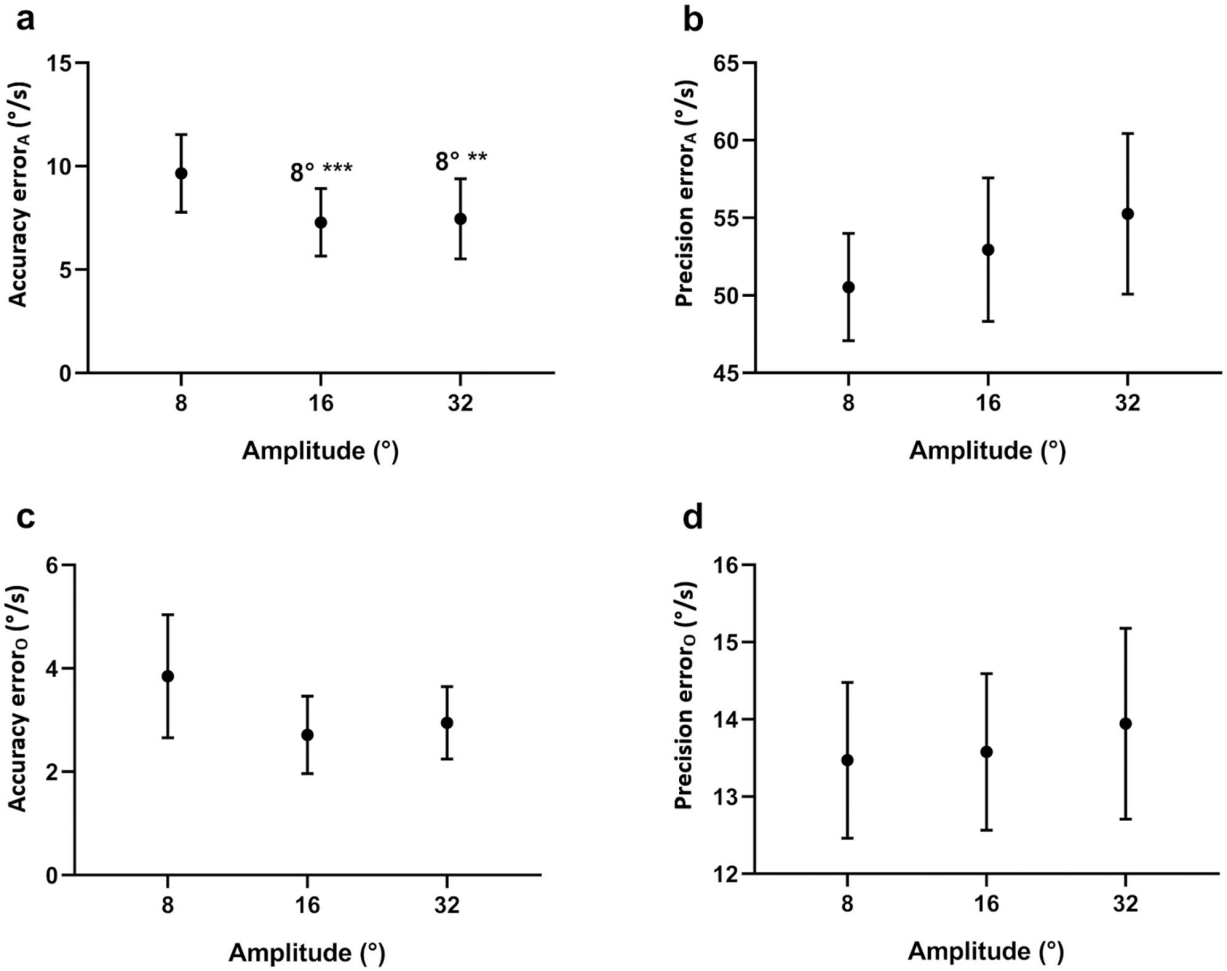
Effect of target amplitude. Data are the mean accuracy and precision error of eye velocity along (**a**, **b**) and orthogonal (**c**, **d**) to the target trajectory. Mean errors for each target amplitude are the result of averaging across all target directions and velocities. The significance of post hoc comparisons is displayed above the data. “8°” indicates those results that were significantly different from 8° target amplitudes. The format and depiction of the levels of statistical significance are the same as Figure 4.

There was a significant main effect for amplitude on accuracy along the target trajectory (*P* < 0.001), with a significantly greater error in accuracy at a target amplitude of 8° than the other, larger amplitudes of 16° or 32° (Fig. 6) (see Supplementary Material S3 for statistics). In other words, accuracy along the target trajectory improved with larger pursuit amplitudes.

#### Target Frequency

**Figure 7.**
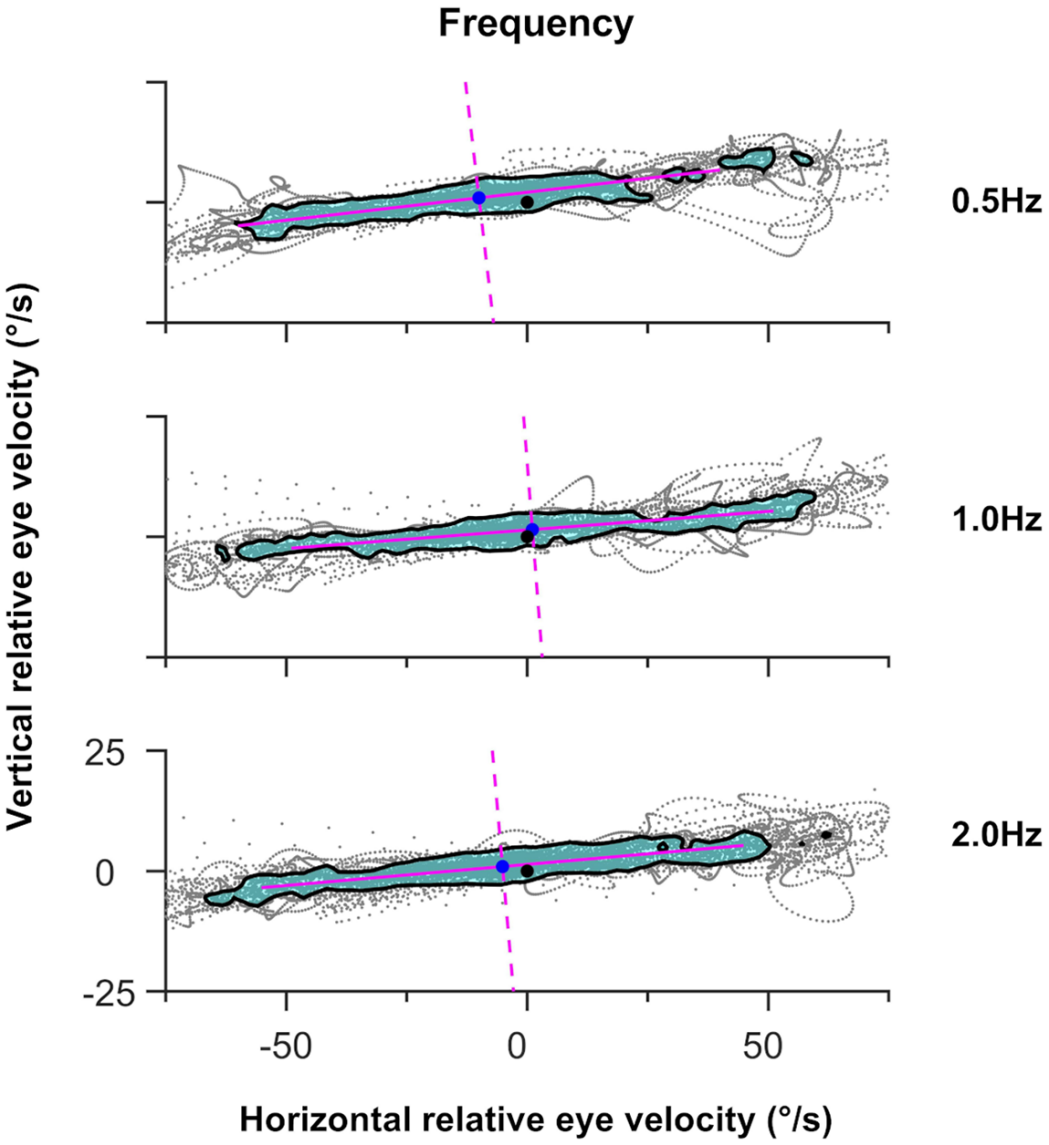
Representative data for target frequency (all data taken from observer 06). The format of each isocontour figure is the same as Figure 2a. Data for the infantile nystagmus cohort indicate greater error in eye velocity accuracy as the target frequency increased.

We reanalyzed our data for 16°/s target motion as a function of target frequency (i.e., target velocity divided by target amplitude) to address the previous claim that those with infantile nystagmus could track rapidly oscillating targets better than typical individuals.^19^ To directly compare our data with Dell'Osso et al.,^19^ we only report data for accuracy and precision along the target trajectory. Representative velocity isocontours for each of the target frequencies (0.5 Hz, 1.0 Hz, and 2.0 Hz) are shown in Figure 7.

**Figure 8.**
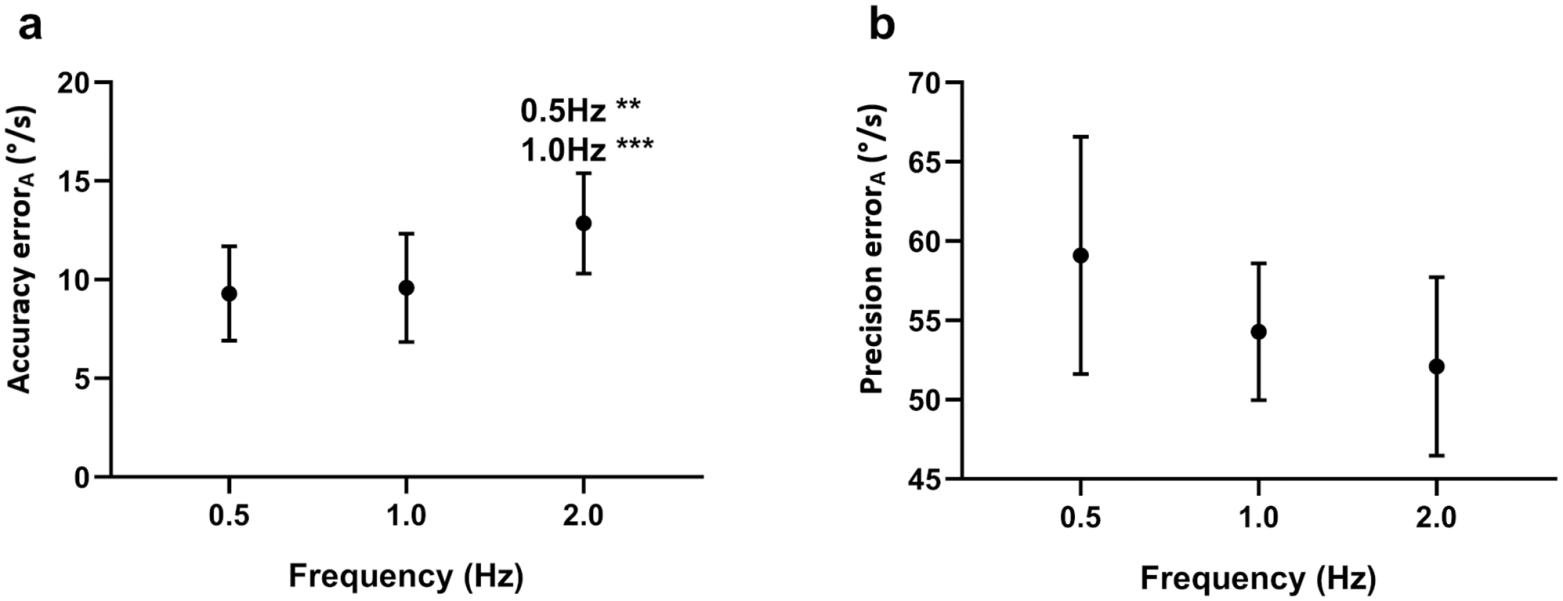
Effect of target frequency. Data are the mean (**a**) accuracy and (**b**) precision error of velocity along the target trajectory. Mean errors for each target frequency are the result of averaging across all target directions. The significance of post hoc comparisons is displayed above the data. “0.5 Hz” and “1.0 Hz” indicate those results that were significantly different from 0.5-Hz and 1.0-Hz target frequencies, respectively. The format and depiction of the levels of statistical significance are the same as Figure 4.

There was a significant main effect for frequency on the accuracy of eye velocity along the target trajectory (*F*(2, 22) = 8.92, *P* = 0.001, ƞ^2^ = .448), with greater error in accuracy at a target frequency of 2.0 Hz than at the other lower frequencies of 1.0 Hz or 0.5 Hz (Fig. 8a) (See Supplementary Material S4 for statistics). There was no significant main effect for frequency on precision along the target trajectory (*P* = 0.106) (Fig. 8b).

## Discussion

Conventionally, pursuit success is quantified by analysis of the eye velocity component parallel to the target. As we have argued, such one-dimensional metrics are inadequate for assessing pursuit performance in those with infantile nystagmus, since both horizontal and vertical eye velocity during pursuit may vary simultaneously. For this reason, we employed a two-dimensional analysis of slow-phase eye velocity to determine the accuracy and precision of pursuit. We acknowledge that the nystagmus slow phase does not represent pure pursuit eye movements per se. However, our aim was to characterize the velocity accuracy and precision with which observers with infantile nystagmus follow moving targets during a pursuit task. In the current study, we have studied vertical pursuit performance in those with infantile nystagmus and reinvestigated previous accounts of horizontal pursuit. Pursuit performance was also quantified as a function of target velocity, amplitude, and frequency.

### Effect of Target Direction

#### Horizontal

Our results for horizontal target motion show that eye velocity was less precise along the axis of the nystagmus oscillation. In other words, horizontal eye velocity was more variable than vertical eye velocity during horizontal pursuit, reflecting the well-established finding that infantile nystagmus is a predominantly horizontal oscillation. Our results reveal that, analogous to an impaired pursuit gain, those with infantile nystagmus have impaired accuracy along the target trajectory during horizontal pursuit. We have previously discussed that the literature for horizontal pursuit in those with infantile nystagmus presents contradictory conclusions; if only the foveation period is analyzed, horizontal pursuit gain is deemed to be normal,^15^^,^^16^ whereas if the entire slow phase is analyzed, then a reduced gain is observed for both horizontal OKN^12^^–^^14^ and horizontal pursuit.^13^^,^^17^ While our data agree with these latter studies that have analyzed the entire slow-phase velocity, we take an alternative interpretation of those former studies analyzing only foveation periods. Since a foveation period immediately follows the end of a corrective fast phase, any relationship between foveation positions with respect to time measures the horizontal positional accuracy of the fast phase in refixating a moving target rather than the underlying pursuit eye velocity (see Supplementary Material S7). Thus, we argue such analyses are not appropriate to be used as the basis for claiming that horizontal pursuit gain in those with infantile nystagmus is “normal.”

#### Vertical

As with horizontal pursuit, we found that eye velocity was less precise along the axis of the nystagmus oscillation, indicating that horizontal eye velocity, as expected, was also more variable than vertical eye velocity during vertical pursuit. In addition, there was impaired accuracy and precision along the target trajectory for vertical pursuit. Indeed, the lack of a significant difference between any of the target directions for accuracy and precision along the target trajectory indicates vertical pursuit was as impaired as horizontal pursuit. This result contrasts with previous qualitative descriptions of vertical pursuit^17^ and quantitative studies of vertical OKN gain.^12^ As discussed, OKN stimuli subtend a large area (42° horizontal × 130° vertically^12^) and so would remain on the fovea despite the lateral movement of the eye arising from the nystagmus oscillation. Thus, studies of OKN are likely to have overestimated pursuit performance in those with infantile nystagmus. When one-dimensional position-time plots of vertical eye position during vertical pursuit are considered (see Fig. 1), the subjective appearance (i.e., smoothness) of pursuit was seemingly quite good, indicating that vertical eye velocity during vertical pursuit is more normal than horizontal eye velocity. However, the target was unlikely to be foveated most of the time. In our study, we employed a small point stimulus typical for pursuit tasks. In this case, observers would be required to pursue the vertical motion of the target extrafoveally, which is likely to have impaired their pursuit performance. Indeed, pursuit of extrafoveal targets has been shown to reduce pursuit gain, with a greater reduction in gain as the eccentricity of the stimulus increased.^27^ Whether those with infantile nystagmus could pursue with high gain if the horizontal position of the target was stabilized with respect to the retina (i.e., gaze contingency) remains to be determined.

### Effect of Target Velocity

When the target velocity increased, the accuracy and precision along the target trajectory decreased, indicating that, like typical individuals,^20^ those with infantile nystagmus struggle to pursue faster targets. In addition, the significant interaction between velocity and direction revealed that vertical pursuit was more impaired by faster targets than horizontal pursuit. During vertical pursuit, it was noted that orthogonal (i.e., horizontal) eye velocity became more variable (lower precision) at a higher target velocity, indicating that velocity of the nystagmus oscillation increased when a faster vertical stimulus was used compared to a slower one. In other words, unlike horizontal pursuit, target speed matters more during vertical pursuit. This may be due to an increased latency to execute a corrective fast phase to a faster moving target, resulting in a longer slow-phase duration that may give rise to larger velocities because the eye is accelerating during the slow phase. Alternatively, a faster vertical pursuit target would be imaged extrafoveally for a longer period than a slower target, potentially further impairing pursuit.

### Effect of Target Amplitude

Decreasing the amplitude of pursuit reduced the accuracy along the target trajectory. This finding may be explained using the results for target frequency, since lowering the amplitude for a given velocity effectively increases the target frequency. When the target frequency is low, there are a greater number of complete nystagmus cycles per sweep. For any given nystagmus cycle where eye velocity is accelerating, the average eye velocity (i.e., intensity) will be low, since eye velocity is biased toward the foveation period. In contrast, when the target frequency is high, there may be only partial nystagmus cycles occurring during any given target sweep. These partial cycles may be of either high or low eye velocity, depending on which aspect (early, mid, or late) of the nystagmus slow phase the target sweep occurred. Thus, this would have lowered the overall accuracy when all sweeps were analyzed collectively. Further, it suggests that the range of eye velocities during higher-frequency target motion would be lower (i.e., a lower error in precision), a trend that we also observed (Fig. 8b), although this was not significant.

### Effect of Target Frequency

Our results for target frequency show impairment of accuracy along the target trajectory at higher target frequencies, contradicting the claim that those with infantile nystagmus can follow a more rapidly oscillating target.^19^ A more plausible alternative explanation for the results of Dell'Osso et al.^19^ is that the pendular oscillation of the single observer studies was, by coincidence, in phase with the target at the higher frequency.

### General Discussion

Our results would lead us to conclude that those with infantile nystagmus inaccurately and imprecisely follow moving targets, whether moving horizontally or vertically, particularly when compared to typical observers (see Supplementary Material S5 and S6). This raises the question of how those with infantile nystagmus follow such moving targets. The fast phase is known to reorient gaze toward the intended target. It has therefore been suggested by others that pursuit in infantile nystagmus is actually saccadic,^17^ with the fast phase redirecting the eye back onto the target, as is the case with microsaccades that occur during fixation of a stationary target.^28^ Indeed, such behavior is not dissimilar to the catchup or backup saccades of typical individuals, especially young children,^29^ during pursuit. In contrast, Dell'Osso et al.^16^ have argued that infantile nystagmus pursuit is not saccadic (i.e., not mediated by fast phases). However, as discussed, foveation periods are essentially a brief extension of the end of the fast phase, indirectly demonstrating that the act of “following” a target is not performed by smooth pursuit eye movements but by an entirely different type of eye movement: fast phases.

### Limitations of the Study

Our study used periodic targets, which are known to be highly predictable in typical observers.^30^ Under these simplified conditions, our results likely represent the upper limit of pursuit performance in those with infantile nystagmus. Indeed, our results for target frequency, which reduced accuracy along the target trajectory, suggest that more complicated patterns of target motion (e.g., random walk of sinusoids)^31^ may result in greater impairment, although fast phases may still be able to supplement pursuit.

The intensity (the product of amplitude and frequency) is a measure of the average velocity of the slow phase. Intensity is known to vary with eye-in-orbit position, and the range of gaze angles with minimum intensity denotes the null zone.^32^^–^^34^ Prior to our experiments, we did not determine the null zone of our observers, so it remains possible that some individuals may have had more intense nystagmus oscillations than others during horizontal pursuit. However, a large cross-sectional study has indicated that the majority (73%) of those with infantile nystagmus have a null zone within ±10° of straight ahead,^6^ suggesting that few of our observers would have had an eccentric null zone. Therefore, horizontal pursuit would usually include the null zone, but this was less likely for vertical pursuit, which was performed at a single horizontal angle.

### Implications

We have taken a novel approach to analyzing pursuit eye movements in those with an eye oscillation by applying those techniques commonly used to quantify the two-dimensional positional stability of fixation in typical individuals.^35^ Our analytical approach also considers the direction of the slow phase (i.e., positive or negative eye velocity). For a moving target, the direction of eye travel may either reduce or increase the retinal image velocity, impacting on the extent of the precision impairment. For example, if the slow phases change direction, this is important information that is not captured using only intensity, the conventional metric for denoting eye velocity precision of the slow phase. To this end, we envisage that the 68% isocontour that bounds the highest probability eye velocities in the bivariate probability density function could also be used to quantify the quality of fixation in those with infantile nystagmus by considering the two-dimensional velocity of the entire slow phase, rather than just the horizontal components of eye velocity (intensity) or arbitrary uniaxial metrics such as foveation period.

During pursuit, the fast phase functions to redirect the fovea back onto the intended target, which itself is moving. Depending on whether the target is moving with or against the nystagmus oscillation, foveation periods may either be extended or reduced, respectively, compared to stationary fixation. However, during vertical target motion, visual discrimination of detail may be impeded further. While some argue for visual acuity in infantile nystagmus to be measured with an unrestricted time limit, under real-world conditions where objects (e.g., people, cars, balls) may move, the abilities of those with infantile nystagmus may, as a result, be overestimated.

We did not specifically undertake an analysis of the fast phases and saccades made by those with infantile nystagmus, since it has been shown by more than one study that they serve a function akin to catchup saccades in reducing the positional error between the target and the eye by reorienting gaze back onto the target. However, of more interest for future studies would be to determine with greater certainty the location of the pursuit target immediately before and after fast phase to determine whether gaze is directed to where the target *was* (i.e., lagging behind the target) or where the target *is* (i.e., intercepting the target).

The presence of an ongoing unidirectional horizontal eye oscillation while attempting to follow vertically moving targets introduces an interesting possibility for trajectory misperception.^36^^,^^37^ In typical individuals, the incomplete compensation of horizontal pursuit-induced retinal image motion by extraretinal signals results in vertically moving targets appearing to move obliquely during horizontal pursuit eye movements.^38^ During the slow phase, those with infantile nystagmus may be susceptible to a similar illusion. However, the extent to which this will impact on pursuit or how well fast phases compensate for any potential error in the perceived trajectory of vertically moving targets is unclear. Future studies could therefore explore the potential influence of the slow-phase velocity on the targeting accuracy of the fast phase.

At least one theory of the mechanism of infantile nystagmus^8^ is founded on the assumption of a normally functioning nystagmus pursuit system. As we have already highlighted, the serious weakness of this theory is that it is underpinned by the claim that those with infantile nystagmus have “normal” pursuit *gain*. Our results question the credibility of such claims, and we argue that more supporting evidence, whether oculomotor or perceptual, is warranted before such a conclusion about pursuit can be supported. For this reason, especially considering the present results, this theory should be interpreted with caution. Nonetheless, based on our findings, we do not claim that the neural substrate of the pursuit system is present or absent, abnormal or normal. Simply, we sought to determine whether the act of pursuing a target recruits eye velocities that are inaccurate and imprecise in those with infantile nystagmus.

If pursuit is the act of stabilizing a small, slowly moving target on the fovea, then contrary to previous claims, we found strong evidence for deficits in both horizontal and vertical pursuit in those with infantile nystagmus. While earlier research has suggested that those with infantile nystagmus can accurately follow a vertically moving OKN target,^12^ these studies mask any potential deficit in the ability to foveate small, vertically moving targets by assuming the fovea is continuously aligned with the target horizontally and the absence of retinal slip. In contrast, our results exposed a substantial deficit in those with infantile nystagmus as they attempt to follow a vertically moving pursuit target. Finally, we found that making the pursuit target oscillate more frequently, even under the simplified condition of predictable image motion, made it more difficult for individuals with infantile nystagmus to track a small moving object.

Visual performance is clinically measured using visual acuity. For those with infantile nystagmus undertaking this test, the fast phase redirects their gaze back onto a *stationary* target. In dynamic environments (e.g., crossing a street with moving traffic, sports), the intended target may not be stationary but moving. Thus, the location of the target at the start of the fast phase may not correspond to the location after the fast phase. If the role of pursuit is to retain a target on the fovea so that it can be scrutinized by higher-resolution retina, then our results suggest current measures may overestimate real-world performance for those with infantile nystagmus.

Pursuit eye movements involve input from the cortex, cerebellum, and brainstem. As such, this class of eye movement represents a measure of the functioning of several important interconnected brain centers (i.e., the integrity of the underlying neural substrate). Our approach of using bivariate probability density functions to quantify performance is undoubtedly more sensitive and informative than the calculation of pursuit gains alone. Such analyses may be more effective in detecting subtle deviations from normal pursuit behavior within other atypical populations and serve as potential biomarkers for assessing rates of disease progression and/or the efficacy of treatments.

## Supplementary Material

Supplement 1

## References

[1] Averbuch-Heller L, Dell'Osso LF, Leigh RJ, Jacobs JB, Stahl JS The torsional component of “horizontal” congenital nystagmus. *J Neuroophthalmol*. 2002; 22: 22–32. 11937902 10.1097/00041327-200203000-00007

[2] Sarvananthan N, Surendran M, Roberts EO, et al. The prevalence of nystagmus: the Leicestershire nystagmus survey. *Invest Ophthalmol Vis Sci*. 2009; 50: 5201–5206. 19458336 10.1167/iovs.09-3486

[3] McLean RJ, Windridge KC, Gottlob I Living with nystagmus: a qualitative study. *Br J Ophthalmol*. 2012; 96: 981–986. 22517800 10.1136/bjophthalmol-2011-301183

[4] Das A, Quartilho A, Xing W, et al. Visual functioning in adults with idiopathic infantile nystagmus syndrome (IINS). *Strabismus*. 2018; 26: 203–209. 30325248 10.1080/09273972.2018.1526958

[5] Bunce C, Zekite A, Wormald R, Bowman R Is there evidence that the yearly numbers of children newly certified with sight impairment in England and Wales has increased between 1999/2000 and 2014/2015? A cross-sectional study. *BMJ Open*. 2017; 7: 1–7. 10.1136/bmjopen-2017-016888 PMC5588960 28864701

[6] Abadi R V, Bjerre A Motor and sensory characteristics of infantile nystagmus. *Br J Ophthalmol*. 2002; 86: 1152–1160. 12234898 10.1136/bjo.86.10.1152 PMC1771304

[7] Leigh RJ, Zee DS Smooth pursuit and visual fixation. In: *The Neurology of Eye Movements*. 3rd ed. New York, NY: Oxford University Press; 1999: 151–197.

[8] Brodsky MC, Dell'Osso LF. A unifying neurologic mechanism for infantile nystagmus. *JAMA Ophthalmol*. 2014; 132: 761–768. 24525626 10.1001/jamaophthalmol.2013.5833

[9] Carpenter RHS *Movements of the Eyes*. 2nd ed. London, UK: Pion Limited; 1988.

[10] Shanidze N, Ghahghaei S, Verghese P Accuracy of eye position for saccades and smooth pursuit. *J Vis*. 2016; 16(15): 23. 10.1167/16.15.23 PMC5213993 28006073

[11] Haarmeier T, Thier P. Impaired analysis of moving objects due to deficient smooth pursuit eye movements. *Brain*. 1999; 122: 1495–1505. 10430833 10.1093/brain/122.8.1495

[12] Abadi R V, Dickinson CM The influence of preexisting oscillations on the binocular optokinetic response. *Ann Neurol*. 1985; 17: 578–586. 4026229 10.1002/ana.410170609

[13] Yamazaki A. Abnormalities of smooth pursuit and vestibular eye movements in congenital jerk nystagmus. In: Oosterhuis J, ed. *Ophthalmology: Proceedings of the XXIII International Congress*. Kyoto: Excerpta Medica, New York; 1978: 1162–1165.

[14] Yee RD, Baloh RW, Honrubia V Study of congenital nystagmus: optokinetic nystagmus. *Br J Ophthalmol*. 1980; 64: 926–932. 7448147 10.1136/bjo.64.12.926 PMC1042576

[15] Dell'Osso LF. Evaluation of smooth pursuit in the presence of congenital nystagmus. *Neuroophthalmology*. 1986; 6: 383–406.

[16] Dell'Osso LF, van der Steen J, Steinman RM, Collewijn H Foveation dynamics in congenital nystagmus. II: Smooth pursuit. *Documenta Ophthalmol*. 1992; 79: 25–49. 10.1007/BF00160131 1568421

[17] Collewijn H, Apkarian P, Spekreijse H The oculomotor behaviour of human albinos. *Brain*. 1985; 108: 1–28. 3978393 10.1093/brain/108.1.1

[18] Kolarik AJ, Margrain TH, Freeman TCA Precision and accuracy of ocular following: influence of age and type of eye movement. *Exp Brain Res*. 2010; 201: 271–282. 19841914 10.1007/s00221-009-2036-6

[19] Dell'Osso LF, Gauthier G, Liberman G, Stark L Eye movement recordings as a diagnostic tool in a case of congenital nystagmus. *Am J Optom Arch Am Acad Optom*. 1972; 49: 3–13. 4500610 10.1097/00006324-197201000-00002

[20] Mcilreavy L, Freeman TCA, Erichsen JT Two-dimensional analysis of smooth pursuit eye movements reveals quantitative deficits in precision and accuracy. *Trans Vis Sci Technol*. 2019; 8(5): 7. 10.1167/tvst.8.5.7 PMC6753966 31588372

[21] Dunn MJ, Harris CM, Ennis FA, et al. An automated segmentation approach to calibrating infantile nystagmus waveforms. *Behav Res Methods*. 2019; 51: 2078–2084. 10.3758/s13428-018-1178-5 PMC6797654 30875024

[22] Imai T, Takimoto Y, Okumura T, et al. Visual target strategies in infantile nystagmus patients with horizontal jerk waveform. *Front Neurol*. 2018; 9: 622. 30104998 10.3389/fneur.2018.00622 PMC6077220

[23] Botev ZI, Grotowski JF, Kroese DP Kernel density estimation via diffusion. *Ann Stat*. 2010; 38: 2916–2957.

[24] Berens P, Valesco MJ. *The Circular Statistics Toolbox for MATLAB*. Tübingen, Germany: Max Planck Institute for Biological Cybernetics; 2009.

[25] Cousineau D. Confidence intervals in within-subject designs: a simpler solution to Loftus and Masson's method. *Tutorials Quantitative Methods Psychol*. 2005; 1: 42–55.

[26] Morey RD. Confidence intervals from normalized data: a correction to Cousineau (2005). *Tutorials Quantitative Methods Psychol*. 2008; 4: 61–64.

[27] Wyatt HJ, Pola J, Fortune B, Posner M Smooth pursuit eye movements with imaginary targets defined by extrafoveal cues. *Vis Res*. 1994; 34: 803–820. 8160395 10.1016/0042-6989(94)90218-6

[28] Dell'Osso LF, van der Steen J, Steinman RM, Collewijn H Foveation dynamics in congenital nystagmus, I: fixation. *Documenta Ophthalmol*. 1992; 79: 1–23. 10.1007/BF00160130 1568420

[29] Vinuela-Navarro V, Erichsen JT, Williams C, Woodhouse JM Effect of stimulus type and motion on smooth pursuit in adults and children. *Optom Vis Sci*. 2017; 94: 760–769. 28609416 10.1097/OPX.0000000000001090

[30] Barnes GR, Asselman PT. The mechanism of prediction in human smooth pursuit eye movements. *J Physiol*. 1991; 439: 439–461. 1895243 10.1113/jphysiol.1991.sp018675 PMC1180117

[31] Lisberger SG, Evinger C, Johanson GW, Fuchs AF Relationship between eye acceleration and retinal image velocity during foveal smooth pursuit in man and monkey. *J Neurophysiol*. 1981; 46: 229–249. 7264712 10.1152/jn.1981.46.2.229

[32] Dell'Osso LF, Flynn JT, Daroff RB Hereditary congenital nystagmus: an intrafamilial study. *Arch Ophthalmol*. 1974; 92: 366–374. 4429465 10.1001/archopht.1974.01010010378002

[33] Abel LA, Daroff RB, Dell'Osso LF Horizontal pursuit defect nystagmus. *Ann Neurol*. 1979; 5: 449–452. 313750 10.1002/ana.410050508

[34] Abadi R V, Whittle J The nature of head postures in congenital nystagmus. *Arch Ophthalmol*. 1991; 109: 216–220. 1993030 10.1001/archopht.1991.01080020062044

[35] Cherici C, Kuang X, Poletti M, Rucci M Precision of sustained fixation in trained and untrained observers. *J Vis*. 2012; 12(6): 1–16. 10.1167/12.6.31 PMC3489479 22728680

[36] Filehne W. Über das optische Wahrnehmen von Bewegungen. *Zeitschrift Sinnesphysiol*. 1922; 53: 134–144.

[37] Freeman T, Banks MS. Perceived head-centric speed is affected by both extra-retinal and retinal errors. *Vis Res*. 1998; 38: 941–945. 9666976 10.1016/s0042-6989(97)00395-7

[38] Souman JL, Hooge ITC, Wertheim AH Vertical object motion during horizontal ocular pursuit: compensation for eye movements increases with presentation duration. *Vis Res*. 2005; 45: 845–853. 15644225 10.1016/j.visres.2004.10.010

