# Supplementary material for: Two-Dimensional Analysis of Horizontal and Vertical Pursuit in Infantile Nystagmus Reveals Quantitative Deficits in Accuracy and Precision
Source: Invest Ophthalmol Vis Sci. 2020 Jun 11;61(6):15. doi: 10.1167/iovs.61.6.15 (PMC7415281; doi:10.1167/iovs.61.6.15)
Supplement: Supplement 1 [file iovs-61-6-15_s001.pdf]

Supplementary Material

Two-dimensional analysis of horizontal and vertical pursuit in infantile nystagmus reveals quantitative deficits in accuracy and precision

Lee Mcilreavy, Tom C.A. Freeman, and Jonathan T. Erichsen

Index

S1 Effect of target direction: descriptive statistics and post hoc comparisons........................2

S2 Effect of target velocity: descriptive statistics and post hoc comparisons..........................3

S3 Effect of target amplitude: descriptive statistics and post hoc comparisons......................4

S4 Effect of target frequency: descriptive statistics and post hoc comparisons......................5

S5 Between-groups ANOVA: observers with infantile nystagmus and typical observers.........6

S6 Between-groups ANOVA: descriptive statistics and ANOVA results 7

S7 Horizontal eye position data for an observer with infantile nystagmus during pursuit 8

S8 References..9

S1 Effect of target direction: descriptive statistics and post hoc comparisons

	Rightward (*°/s ± 95%CI*)	*Leftward* (*°/s ± 95%CI*)	*Upward* (*°/s ± 95%CI*)	*Downward* (*°/s ± 95%CI*)
Accuracy $_A$	7.33 ± 1.63	12.11 ± 2.47	6.41 ± 1.43	6.67 ± 1.60
Precision $_A$	54.96 ± 5.39	56.58 ± 4.99	49.17 ± 4.22	50.94 ± 4.50
Accuracy $_O$	1.53 ± 0.53	1.37 ± 0.50	5.05 ± 1.30	4.70 ± 1.05
Precision $_O$	11.34 ± 0.96	12.38 ± 1.25	15.43 ± 1.18	15.50 ± 1.09

Descriptive statistics for accuracy and precision of pursuit along (subscript A) and orthogonal (subscript O) to the target trajectory as a function of target direction. Data are mean values ± 95% confidence intervals.

Comparison		*t*	p_{bonf}
Rightward	*Leftward*	0.77	1.00
	Upward	-3.27	**.045**
	Downward	3.34	**.039**
Leftward	*Upward*	-3.51	**.030**
	Downward	-3.64	**.023**
Upward	*Downward*	0.04	1.00

Post hoc statistical comparisons for eye velocity accuracy orthogonal to target trajectory as a function of target direction. Statistically significant comparisons are shown in bold.

Comparison		*t*	p_{bonf}
Rightward	*Leftward*	-1.69	.718
	Upward	-3.55	**.027**
	Downward	-3.37	**.037**
Leftward	*Upward*	-1.99	.427
	Downward	-2.01	.421
Upward	*Downward*	-0.14	1.00

Post hoc statistical comparisons for eye velocity precision orthogonal to target trajectory as a function of target direction. Statistically significant comparisons are shown in bold.

S2 Effect of target velocity: descriptive statistics and post hoc comparisons

	8°/s (*°/s ± 95%CI*)	*16°/s* (*°/s ± 95%CI*)
Accuracy $_A$	5.68 ± 1.52	10.58 ± 1.61
Precision $_A$	50.67 ± 7.28	55.16 ± 4.06
Accuracy $_O$	2.97 ± 0.82	3.37 ± 0.97
Precision $_O$	12.72 ± 0.76	14.61 ± 1.27

Descriptive statistics for accuracy and precision of pursuit along (subscript A) and orthogonal (subscript O) to the target trajectory as a function of target velocity. Data are mean values ± 95% confidence intervals.

Comparison		*t*	p_{bonf}
8°/s	*16°/s*	-8.51	**<.001**

Post hoc statistical comparison for eye velocity accuracy along the target trajectory as a function of target velocity. Bold indicates a statistically significant result.

Comparison		*t*	p_{bonf}
8°/s	*16°/s*	-3.06	**.011**

Post hoc statistical comparison for eye velocity precision along the target trajectory as a function of target velocity. Bold indicates a statistically significant result.

Comparison		*t*	p_{bonf}
8°/s	*16°/s*	-4.51	**<.001**

Post hoc statistical comparison for eye velocity precision orthogonal to the target trajectory as a function of target velocity. Bold indicates a statistically significant result.

S3 Effect of target amplitude: descriptive statistics and post hoc comparisons

	8° (*°/s ± 95%CI*)	*16°* (*°/s ± 95%CI*)	*32°* (*°/s ± 95%CI*)
Accuracy $_A$	9.65 ± 1.87	7.29 ± 1.63	7.46 ± 1.93
Precision $_A$	50.53 ± 3.46	52.94 ± 4.62	55.26 ± 5.18
Accuracy $_O$	3.84 ± 1.19	2.71 ± 0.75	2.95 ± 0.70
Precision $_O$	13.47 ± 1.01	13.58 ± 1.01	13.94 ± 1.23

Descriptive statistics for accuracy and precision of pursuit along (subscript A) and orthogonal (subscript O) to the target trajectory as a function of target amplitude. Data are mean values ± 95% confidence intervals.

Comparison		*t*	p_{bonf}
8°	*16°*	-5.60	**<.001**
	32°	-3.17	**.027**
16°	*32°*	0.27	1.00

Post hoc statistical comparisons for eye velocity accuracy along the target trajectory as a function of target amplitude. Statistically significant comparisons are shown in bold.

S4 Effect of target frequency: descriptive statistics and post hoc comparisons

	0.5Hz (°/s ± 95%CI)	*1.0Hz (°/s ± 95%CI)*	*2.0Hz (°/s ± 95%CI)*
Accuracy $_A$	9.29 ± 2.39	9.59 ± 2.75	12.85 ± 2.54
Precision $_A$	59.10 ± 7.48	54.28 ± 4.32	52.09 ± 5.63

Descriptive statistics for accuracy and precision of pursuit along (subscript A) and orthogonal (subscript O) to the target trajectory as a function of target frequency. Data are mean values ± 95% confidence intervals.

Comparison		*t*	p_{bonf}
0.5Hz	*1.0Hz*	-0.30	1.00
	2.0Hz	-3.12	**.029**
1.0Hz	*2.0Hz*	-5.58	**<.001**

Post hoc statistical comparisons for eye velocity accuracy along the target trajectory as a function of target frequency. Statistically significant comparisons are shown in bold.

S5 Between-groups ANOVA: observers with infantile nystagmus and typical observers

We have previously undertaken a study of pursuit performance in typical observers using an identical protocol [1]. A between-groups ANOVA showed a significant main effect of group on the accuracy and precision of eye velocity along (accuracy, $F(1,25)=33.62$, $p<.001$, $\eta^2=.574$; precision, $F(1,25)=29.20$, $p<.001$, $\eta^2=.539$) and orthogonal (accuracy, $F(1,25)=25.22$, $p<.001$, $\eta^2=.502$; precision, $F(1,25)=44.50$, $p<.001$, $\eta^2=.641$) to the target trajectory, and in all cases, observers with infantile nystagmus had a greater error in accuracy and precision than typical observers (see Supplementary Material S6).

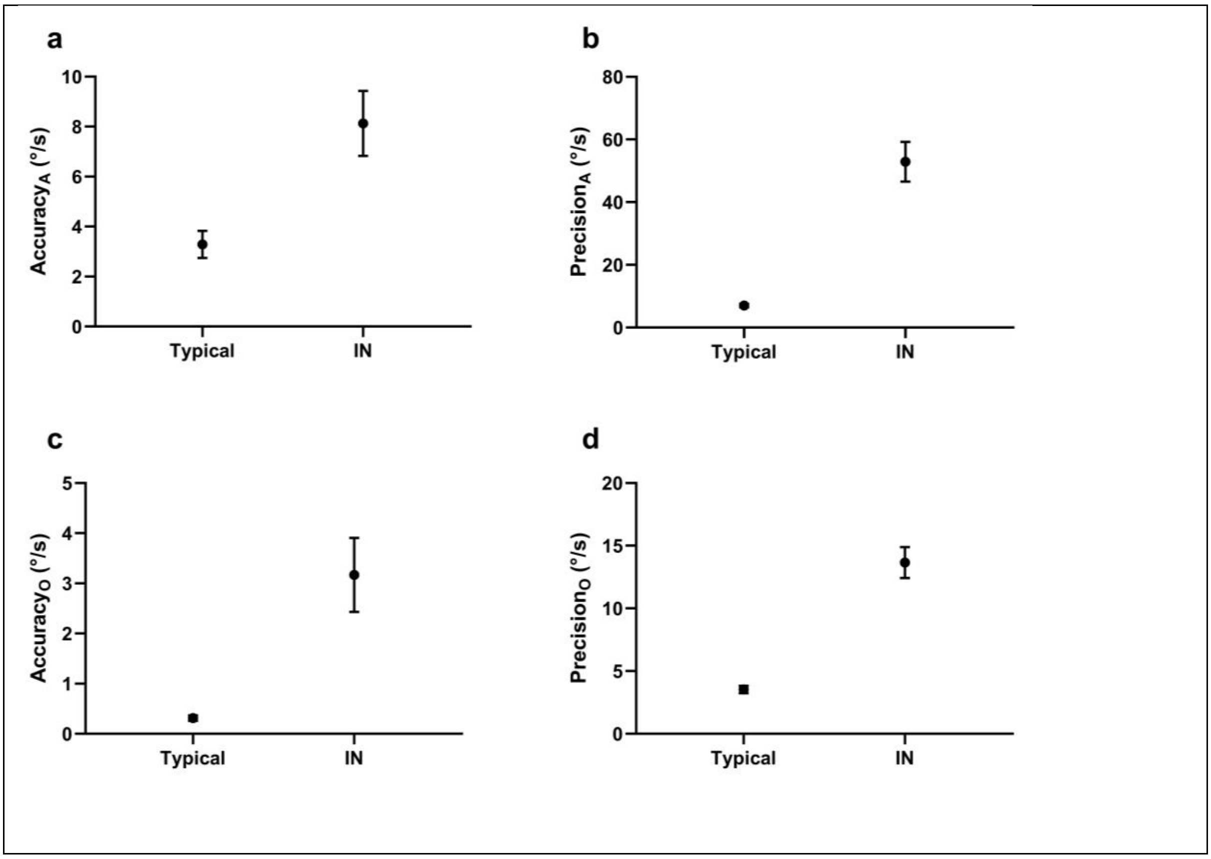

Means of the accuracy and precision errors along (a, b) and orthogonal (c, d) to the target trajectory for typical observers and those with infantile nystagmus. Mean errors for each group are the result averaging across all pursuit trials. Error bars represent the 95% confidence intervals, calculated using the Cousineau-Morey method (Cousineau, 2005; Morey, 2008). The levels of statistical significance are denoted as: *** p < .001; ** p < .010; * p < .050.

S6 Between-groups ANOVA: descriptive statistics and ANOVA results

	Typical (°/s ± 95%CI)	IN (°/s ± 95%CI)
Accuracy $_A$	3.29 ±0.54	8.13 ±1.30
Accuracy $_O$	0.31 ±0.05	3.17 ±0.74
Precision $_A$	7.04 ±0.54	52.91 ±6.35
Precision $_O$	3.54 ±0.29	13.66 ±1.24

Descriptive statistics for accuracy and precision of pursuit along (subscript A) and orthogonal (subscript O) to the target trajectory as a function of group. Data are mean values ± 95% confidence intervals.

	df	*F*	*p*	η^2
Accuracy $_A$	1.00, 25.00	33.62	**<.001**	.574
Accuracy $_O$	1.00, 25.00	25.22	**<.001**	.502
Precision $_A$	1.00, 25.00	29.20	**<.001**	.539
Precision $_O$	1.00,25.00	44.60	**<.001**	.641

Results of a between-groups ANOVA for accuracy and precision of pursuit along (subscript A) and orthogonal (subscript O) to the target trajectory as a function of group. Statistically significant main effects are shown in bold.

S7 Horizontal eye position data for an observer with infantile nystagmus during pursuit

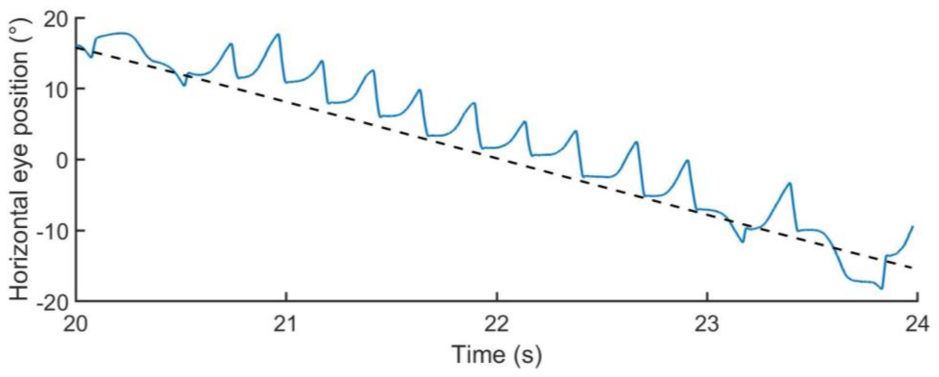

Representative position-time plot of horizontal eye position (blue trace) during horizontal pursuit (data from observer 03). The target trajectory (black trace) was leftward with an amplitude of 36° at 8°/s. In this trace the slow phases can be seen to move gaze away from the target before fast phases redirect gaze back on to the target. Since foveation periods immediately follow the end of the fast phase, we argue that a line of best fit through the foveation positions measures the horizontal accuracy of the fast phases rather than an underlying pursuit velocity, as argued by others [2,3].

S8 References

1. Mcilreavy L, Freeman TCA, Erichsen JT. Two-Dimensional Analysis of Smooth Pursuit Eye Movements Reveals Quantitative Deficits in Precision and Accuracy. *Translational Vision Science & Technology*. 2019;8:7.

2. Dell'Osso LF. Evaluation of smooth pursuit in the presence of congenital nystagmus. *Neuro-Ophthalmology*. 1986;6:383-406.

3. Dell'Osso LF, van der Steen J, Steinman RM, Collewijn H. Foveation dynamics in congenital nystagmus. II: Smooth pursuit. *Documenta Ophthalmologica*. 1992;79:25-49.
